# Novel agents and strategies for overcoming EGFR TKIs resistance

**DOI:** 10.1186/2162-3619-3-2

**Published:** 2014-01-11

**Authors:** Fei-Yu Niu, Yi-Long Wu

**Affiliations:** 1 Guangdong Lung Cancer Institute, Guangdong General Hospital & Guangdong Academy of Medical Sciences, Guangzhou, PR China

**Keywords:** Non-small cell lung cancer, EGFR TKI acquired resistance, New agents, IMPRESS, ASPIRATION

## Abstract

Since the recognition of epidermal growth factor receptor (EGFR) as a therapeutic target, EGFR tyrosine kinase inhibitors (TKIs) have been used in lung cancer patients with EGFR mutations, which has been a major breakthrough for lung cancer treatment.. The progression-free survival (PFS) of patients with EGFR mutations treated with EGFR TKIs is significantly prolonged compared with that of patients who underwent standard chemotherapy. However, all patients who initially respond to EGFR TKIs eventually develop acquired resistance (AR). Many small molecule agents and monoclonal antibodies (McAb) targeting signaling pathways are potential therapeutic regimens for overcoming resistance, and various therapeutic strategies are used in clinical practice. Here we review the novel agents and therapeutic strategies for overcoming AR to EGFR TKIs.

## Introduction

Non-small cell lung cancer (NSCLC) accounts for approximately 80% of all lung cancer cases [1]. The traditional treatment for NSCLC is platinum-based chemotherapy, which inhibits normal and cancer cells, but it has reached a therapeutic plateau [2]. In contrast, personalized therapy has numerous advantages [3]. The ERBB family includes EGFR/HER1, ERBB2/HER2, ERBB3/HER3 and ERBB4/HER4. Somatic EGFR mutations result in conformational changes and the activation of downstream signaling. Gefitinib and erlotinib are first-generation EGFR TKIs that reversibly compete with the ATP-binding site in the EGFR. Gefitinib/erlotinib (G/E) prolongs progression-free survival (PFS) and improves quality of life compared with standard chemotherapy, while almost all patients develop AR to G/E in 9 to 11 months [4].

**Figure 1:**
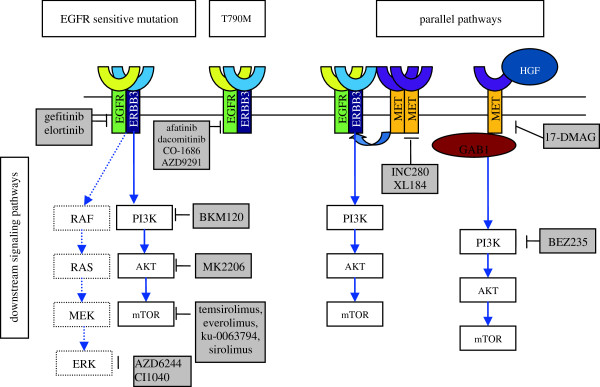
**New agents for overcoming AR.** This figure shows the parallel and downstream signaling pathways of the EGFR. The gray boxes depict new agents available for treatment.

The mechanisms responsible for AR to G/E include the following: first, secondary EGFR mutation, which is predominantly T790M; second, activation of parallel signaling pathways; third, activation of downstream signaling pathways; fourth, phenotypic transformation, such as epithelial to mesenchymal transition (EMT) and small cell lung cancer (SCLC) transformation. We will discuss the mechanisms of AR to G/E and corresponding novel targeting agents (Figure 1). The potential therapeutic options for patients with AR to EGFR TKIs are also reviewed.

## New agents for overcoming AR

### Secondary EGFR mutations

The most common type of AR is mutation in codon 790 (T790M) in EGFR exon 20, which accounts for 50 ~ 60% of cases with AR to G/E [5]. EGFR T790M leads to a higher affinity of ATP for EGFR, thus affecting G/E binding [6]. Second generation EGFR TKIs e.g., BIBW 2992 (afatinib) and PF00299804 (dacomitinib), and third generation EGFR TKIs e.g., CO-1686 and AZD9291 are irreversible inhibitors that could overcome the AR caused by T790M. Afatinib has been approved by the FDA. Dacomitinib, CO-1686 and AZD9291 are currently being tested in ongoing trials.

#### Afatinib

The safety and efficacy of afatinib have been characterized in many clinical trials. The most classic trial is the LUX-Lung program. This program evaluated the efficacy of afatinib for first-line treatment and for patients who have been treated with G/E. The efficacy of afatinib in overcoming AR to G/E was confirmed in the LUX-Lung 1/4/5 trials.

A total of 585 patients who had received G/E and chemotherapy were enrolled in LUX-Lung 1 [7], and these patients were randomized to receive afatinib or placebo. The primary endpoint, overall survival (OS), was 10.8 months in the afatinib group, while the outcome was 12.0 months in placebo group (HR = 1.077, P = 0.74). However, PFS was significantly prolonged in the afatinib group (3.3 vs. 1.1 months, HR = 0.38; p < 0.0001). Moreover, symptoms such as cough and dyspnea were significantly improved in the afatinib group.

LUX-Lung 4 [8] also confirmed the efficacy of afatinib in patients who progressed on G/E and chemotherapy. In the 61 evaluable patients, partial response (PR) was achieved in 4 patients, and the median PFS and OS was 4.4 and 19.0 months, respectively.

Afatinib was investigated in patients who failed chemotherapy and G/E in LUX-Lung 5 [9]. The patients were randomized into two groups after afatinib resistance i.e., afatinib alone or afatinib combined with paclitaxol. In part A of the trial, 63% of the patients achieved complete response (CR)/PR/stable disease (SD), and the median PFS was 3.3 months. The results of part B are pending.

#### Dacomitinib

In a phase II trial, dacomitinib was given to patients with advanced NSCLC after chemotherapy (CT) and erlotinib (E) failure. In 62 evaluable cases, 3 had a partial response, and 35 had stable disease (SD) for more than 6 weeks. The side effects included diarrhea (86%), fatigue (40%), rash (45%), and stomatitis/mucosal inflammation, which was mainly grade 1/2 and manageable [10]. One patient with a T790M mutation caused by prior gefitinib treatment achieved SD after exposure to dacomitinib in the NCT00783328 trial [11]. Four patients who have failed G/E achieved PR in a phase I trial (NCT00225121) [12]. NCT01000025 is an ongoing phase III trial that investigates the efficacy of dacomitinib in patients with NSCLC who have failed chemotherapy and G/E.

#### CO-1686 and AZD9291

CO-1686 is a third-generation EGFR TKI, which may be more effective in patients with T790M. NCT01526928 is an ongoing phase I/II trial that has enrolled patients who have failed prior chemotherapy and EGFR-targeted therapy including erlotinib, gefitinib, neratinib, afatinib, or dacomitinib. Three cases treated with CO-1686 demonstrated clinical benefit or tumor shrinkage, and the tolerance was acceptable [13].

AZD9291 is a potent oral, irreversible inhibitor of the *EGFR* with EGFR-TKI-sensitizing (*EGFR*+) and resistance mutations (T790M). The AZD9291 IC_50_ for the *EGFR*+/T790M H1975 cells is 15 nM. In a phase I open-label multicenter study, AZD9291 was shown to have mostly mild AEs, and no grade 3-4 SAEs were observed. Moreover, 89 patients received at least one dose, and no DLTs were observed. Good evidence for efficacy has been observed at all doses studied thus far, including 9/18 patients with T790M who had confirmed or unconfirmed partial responses [14].

### Parallel pathway activation

#### MET amplication/overexpression

The incidence of MET gene amplication or protein overexpression is 5 ~ 22% in AR patients [15]. MET activates ERBB3 and the PI3K/AKT pathway independent of EGFR [16]. MetMAb [17] and ARQ197 (tivantinib) [18] are the most prominent MET inhibitors, but they have not been investigated for overcoming TKI resistance.

NCT01610336 and NCT01911507 are ongoing trials that test the safety and efficacy of INC280 in NSCLC patients with AR to G/E. INC280 and gefitinib are simultaneously given to patients with MET-amplification in the NCT01610336 trial. Patients with MET overexpression are treated with INC280 and erlotinib in the NCT01911507 trial. The results of these trials are not yet available.

XL184 is a tyrosine kinase inhibitor of multiple receptors, including VEGFR2, MET, and RET. NCT00596648 is a phase Ib/II trial evaluating the safety and efficacy of XL184 with or without erlotinib in patients resistant to EGFR TKIs, and its results are pending.

#### HGF overexpression

Approximately 60% patients with AR to G/E have HGF overexpression [19]. The overexpression results in MET phosphorylation and the activation of GAB1 and PI3K/AKT [20]. BEZ235 is PI3K-mTOR inhibitor that has the potential to overcome AR in vitro [21]. However, the efficacy of BEZ235 has not been tested in vivo. Heat shock protein 90 (HSP90) is a molecular chaperone for several proteins, including EGFR and MET. 17-DMAG is an HSP90 inhibitor that has efficacy for HGF-triggered erlotinib resistance in cell lines and animal models [22]. AUY922 is also an HSP90 inhibitor that is currently being tested in the phase II trial NCT01259089. This trial enrolled patients with AR to G/E. The ORR was 13%, and the two patients with a PR had an EGFR T790M mutation [23].

### Downstream signaling pathway activation

The downstream signaling pathways of EGFR include the RAS/RAF/MEK/ERK and PI3K/AKT/mTOR pathways. The former is associated with proliferation, and the latter is related to survival. Mutations in EGFR result in activation of the PI3K/AKT pathway and the survival of tumor cells without affecting tumor cell proliferation. The mutation of key genes in these two pathways leads to G/E resistance.

#### PIK3CA mutation

PIK3CA mutations were detected in 5% of patients with AR to G/E [24]. The most promising PI3K inhibitor is BKM120, which is a pan-PI3K inhibitor. The antitumor activation of gefitinib plus BKM120 was observed in patients with AR to G/E in the phase Ib trial NCT01570296 [25]. A reduction in SUVmax (>25%) was observed in 4/10 patients, and the median PFS was 2.8 months. Given the favorable central nervous system (CNS) penetration of BKM120, patients with brain metastases were included, and two patients with CNS penetration had a PFS of 2.8 and 10.7 months. However, molecular analysis revealed that no patient harbored a PIK3CA mutation. NCT01487265 is an ongoing phase I/II trial examines the efficacy of BKM120 combined with erlotinib in patients with AR to erlotinib, and the results of this trial are pending.

#### AKT

AKT mutations were not detected in patients with AR to G/E [26], but AKT might be activated in tumors resistant to G/E. MK2206 is one of the most potent AKT inhibitors. Growth inhibition was greatly enhanced with the combination of MK2206 and erlotinib in TKI-sensitive and TKI-resistant NSCLC cell lines, and MK-2206 restored erlotinib sensitivity in HGF-induced AR cells [27]. There are two ongoing trials, NCT01294306 and NCT01147211, which examine the efficacy of MK2206 in NSCLC patients with AR to erlotinib and gefitinib, respectively, but their results are unavailable.

#### mTOR

mTOR regulates cell growth and metabolism by controlling several catabolic and anabolic processes. mTOR inhibitors include temsirolimus, everolimus (RAD001), Ku-0063794, and sirolimus.

Temsirolimus and everolimus could overcome HGF-induced EGFR TKI AR in vitro [28]. Everolimus enhanced the antitumor activation of gefitinib in sensitive and resistant cell lines [29] but failed to meet the primary endpoint in a phase II trial [30]. Ku-0063794 could effectively inhibit gefitinib-resistant cells [31]. NCT00993499 is an ongoing phase Ib trial in which the efficacy of the combination of afatinib and sirolimus is being tested in patients with AR to gefitinib or erlotinib, and the results are pending.

### ERK2 amplification

ERK2 (MAPK1) amplification has been reported in 5% of patients with AR to G/E [32]. AZD6244 and CI1040 could reverse resistance in cells with AR to gefitinib [33]; however, there is no clinical trial that has evaluated the efficacy of overcoming AR to AZD6244 and CI1040.

## Strategies for overcoming AR

### IMPRESS model

Some patients may have accelerated disease progression after discontinuing G/E during washout periods. This phenomenon is called disease flare, which was observed in 23% of patients with AR [34]. To avoid disease flare, some clinicians suggest G/E continuation plus chemotherapy. There was a retrospective study demonstrating that erlotinib continuation in addition to chemotherapy significantly improved the respond rate (RR) compared with chemotherapy alone after erlotinib failure [35]. IMPRESS is an ongoing phase III trial enrolling patients with EGFR mutations who failed first-line gefitinib. The patients are randomized to receive cisplatin and pemetrexed plus gefitinib or placebo. The primary endpoint of this study is PFS, and the results are pending.

### ASPIRATION model

As EGFR TKIs greatly improve the PFS of patients with EGFR positive mutations, the RECIST definition of progression now appears to be challenged. Recently Yang et al. reported a retrospective study that divided patients with AR into three groups: dramatic progression, local progression and gradual progression. The authors recommended chemotherapy for patients with dramatic progression, the continuation of TKIs plus local intervention for those with local progression and the continuation of TKIs for those with gradual progression [36]. ASPIRATION is an ongoing phase II study evaluating the continuation of erlotinib beyond RECIST PD in selected patients who have slowed PD (>6 months of partial response/stable disease), asymptomatic minimal PD, or new, locally controlled brain metastases.

### Switching to or combining with novel compounds

With the unveiling of the underlying mechanisms of AR, novel small molecule inhibitors alone or in combination will be effective in overcoming AR e.g., INC280 plus gefitinib or erlotinib as mentioned above. Therefore, we should recommend that patients receive rebiopsies after progression on G/E to identify the underlying mechanisms of AR. Based on the understanding of resistance mechanisms, clinicians should encourage patients to enter clinical trials.

## Conclusion

EGFR TKIs are increasingly used in the clinic. The standard treatment for patients with AR to EGFR TKIs is changing to chemotherapy at RECIST progression. However, this standard treatment is now being challenged due to a growing number of effective novel compounds and clinical trial strategies.

## Abbreviations

EGFR: Epidermal growth factor receptor; TKIs: Tyrosine kinase inhibitors; PFS: Progression-free survival; AR: Acquired resistance; McAb: Monoclonal antibodies; G/E: Gefitinib/erlotinib; EMT: Epithelial mesenchymal transition; SCLC: Small cell lung cancer; OS: Overall survival; PR: (Partial response); CR: Complete response; SD: Stable disease; HSP90: Heat shock protein 90; RR: Response rate.

## Competing interests

The authors have no relevant conflicts of interests.

## Authors’ contributions

Both authors were involved in preparing, drafting and revising the manuscript, and both have read and approved the final version.
